# Endogenous Mouse Mammary Tumor Viruses (*Mtv*): New Roles for an Old Virus in Cancer, Infection, and Immunity

**DOI:** 10.3389/fonc.2013.00287

**Published:** 2013-11-26

**Authors:** Michael P. Holt, Ethan M. Shevach, George A. Punkosdy

**Affiliations:** ^1^Laboratory of Immunology, National Institute of Allergy and Infectious Diseases, National Institutes of Health, Bethesda, MD, USA

**Keywords:** endogenous retrovirus, mouse mammary tumor virus, immune system, infection, cancer

## Abstract

Mouse Mammary Tumor Viruses are beta-retroviruses that exist in both exogenous (MMTV) and endogenous (*Mtv*) forms. Exogenous MMTV is transmitted via the milk of lactating animals and is capable of inducing mammary gland tumors later in life. MMTV has provided a number of critical models for studying both viral infection as well as human breast cancer. In addition to the horizontally transmitted MMTV, most inbred mouse strains contain permanently integrated *Mtv* proviruses within their genome that are remnants of MMTV infection and vertically transmitted. Historically, *Mtv* have been appreciated for their role in shaping the T cell repertoire during thymic development via negative selection. In addition, more recent work has demonstrated a larger role for *Mtv* in modulating host immune responses due to its peripheral expression. The influence of *Mtv* on host response has been observed during experimental murine models of Polyomavirus- and ESb-induced lymphoma as well as *Leishmania major* and *Plasmodium berghei* ANKA infection. Decreased susceptibility to bacterial pathogens and virus-induced tumors has been observed among mice lacking all *Mtv*. We have also demonstrated a role for *Mtv* Sag in the expansion of regulatory T cells following chronic viral infection. The aim of this review is to summarize the latest research in the field regarding peripheral expression of *Mtv* with a particular focus on their role and influence on the immune system, infectious disease outcome, and potential involvement in tumor formation.

## Introduction

The integration of viral nucleic acid sequences into the host genome is a hallmark of the retroviral life cycle. Integration in somatic cells results in infection of the host and transmission of the virus requires the production of infectious viral particles that are passed on to a new host horizontally. On the other hand, integration in the germ line results in the generation of endogenous retroviruses that become an inheritable part of the host genome. Endogenous retroviruses constitute a significant fraction of various vertebrate genomes, including both human and mouse (1–3). Two of the more widely studied endogenous retroviruses within the mouse genome are the murine leukemia virus (4) and the mouse mammary tumor virus (5), which exist in both exogenous (MMTV) and endogenous (*Mtv*) forms. While the exogenous MMTV has been extensively studied for its role in the establishment and transmission of mammary carcinomas (6, 7), much less remains known about the influence of the endogenous *Mtv* on the host. The aim of this review is to focus on recent advances in understanding the role of endogenous *Mtv*, particularly in relation to cancer, infection, and immunity.

Mice inherit *Mtv* that have integrated into the host genome according to Mendelian inheritance patterns. Greater than 30 different endogenous *Mtv* have been identified (8). The most common inbred laboratory mice contain between two and eight copies of endogenous *Mtv*, the majority of which are shared between several different strains (Table 1) (5, 9). Certain wild-derived (feral) strains of mice, including PERA/Ei and Czech II, are completely devoid of endogenous *Mtv*. Only a few of the endogenous *Mtv*, including *Mtv-1, -2*, and *-4* have retained the ability of forming infectious viral particles (10, 11). Some proviruses, including *Mtv-2*, are capable of both exogenous and endogenous transmission (12).

**Table 1:** **Expression of endogenous *Mtv* among common mouse strains**.

Mouse strain	*Mtv provirus*	H-2	I-A	I-E
A/J	6, 8, 13	H-2^a^	I-A^k^	I-E^k^
AKR/J	7, 8, 9, 17, 23, 30	H-2^k^	I-A^k^	I-E^k^
BALB/cJ	6, 8, 9	H-2^d^	I-A^d^	I-E^d^
C3H/HeJ	1, 6, 8, 11, 14	H-2^k^	I-A^k^	I-E^k^
C57BL/6J	8, 9, 17	H-2^b^	I-A^b^	Null
C58/J	3, 7, 17	H-2^k^	I-A^k^	I-E^k^
CBA/CaJ	8, 9, 14	H-2^k^	I-A^k^	I-E^k^
CBA/J	6, 7, 8, 9, 14, 17	H-2^k^	I-A^k^	I-E^k^
DBA/2J	1, 6, 7, 8, 11, 13, 14, 17	H-2^d^	I-A^d^	I-E^d^
SJL/J	8, 29, 31	H-2^S^	I-A^s^	Null

Endogenous *Mtv* maintain a genetic structure similar to their exogenous MMTV counterparts. For a detailed description of this genetic makeup see the review by Ross (13). Briefly, MMTV is a type B retrovirus of the *Retroviridae* family that contains a 9 kb RNA genome encoding virion capsid (Gag) proteins, reverse transcriptase and integrase enzymes necessary for viral replication (Pol), and envelope (Env) proteins used for viral entry. Like all other retroviruses, MMTV is flanked by 5′ and 3′ long terminal repeats (LTRs). The 3′ LTR of MMTV contains an open reading frame that encodes the viral accessory protein, superantigen (Sag), a type 2 transmembrane glycoprotein (14, 15). More recent data has demonstrated that the 3′ LTR of MMTV also encodes another accessory protein, regulatory of export of MMTV (Rem). Rem is required for efficient nuclear export of unspliced viral RNA via interaction with a Rem-responsive element present in MMTV RNA (16). Both Sag and Rem are encoded by alternatively spliced mRNAs. Rem is related to the human immunodeficiency virus (HIV) Rev protein, thus making MMTV a complex retrovirus (17, 18). Endogenous *Mtv* have accumulated various point mutations or deletions in their proviral genome (19–21). However, almost all *Mtv* have maintained functional Sag expression. While other viral proteins play important roles (especially in terms of viral particle assembly for MMTV), it is the expression of Sag by both MMTV and *Mtv* that has been the most extensively studied. Sag expression plays an important role in the biology of both forms of the virus that is best understood from a historical perspective.

### Importance of Sag in exogenous MMTV infection

As early as 1936, it was observed that certain strains of inbred mice at Jackson Laboratories (ME, USA) displayed an inherent susceptibility to spontaneous mammary carcinomas. The incidence of tumor development ranged from high, intermediate, and low depending on the particular strain of mouse (22). It became readily apparent that the cancer-inducing agent was maternally transmitted and present in milk, and subsequent experiments demonstrated that the causative agent was a virus. Further work led to the discovery and identification of MMTV, originally known as Bittner virus, as the causative agent (23).

In order for MMTV to reach the mammary gland, a complex series of events must occur in which the virus requires and subverts cells of the immune system, particularly B and T cells, to establish infection. The initial site of milk-borne MMTV infection is the gut-associated lymphoid tissue, specifically the Peyer’s patches, where B cells represent the initial target cell (24). The initial round of B cell infection and activation occurs as a result of the interaction between MMTV Env protein and Toll-like receptor 4 (25). The ability of MMTV to disseminate from the gut to the mammary gland is dependent upon this expression of the virally encoded Sag (26). Infected B cells present Sag in conjunction with the major histocompatibility complex (MHC) class II proteins to CD4^+^ T cells bearing a reactive T cell receptor (TCR) Vβ chain. A polymorphic region within the carboxyl terminus of MMTV Sag determines the TCR Vβ domain specificity (27–29). Different MHC class II alleles display strikingly disparate Sag presentation capabilities, with MHC class II I-E inducing the most efficient presentation to T cells (30). Stimulation of Sag-reactive CD4^+^ T cells leads to their activation and production of various cytokines and chemokines. In addition, activated T cells upregulate CD40 ligand (CD40L), which binds to the CD40 receptor on B cells to further activate them (31). Such activation stimulates and recruits additional B and T cells, thereby resulting in further immune cell activation and the subsequent amplification of a reservoir of MMTV infected cells (32). Infected B cells then travel to the developing mammary gland and thereby enable the virus to infect the tumor-susceptible target organ. The critical requirement for an infected lymphocyte population during MMTV infection and dissemination is demonstrated by the resistance of nude mice which lack a functional T cell compartment (33), neonatal thymectomized mice (34), mice lacking Sag-reactive T cells (26), or B cell deficient mice (24) to milk-borne transmission of MMTV-induced tumor development. Mice with inefficient presentation of MMTV Sag due to MHC class II mutations are also resistant to viral infection (35).

Since MMTV does not encode an oncogene, mammary tumorigenesis occurs after insertion of proviral DNA near cellular proto-oncogenes and activation of transcription (36–38). Analysis of genes activated by integration of the MMTV provirus using the viral genome as a molecular tag enabled the identification of a number of MMTV-tagged genes. Of these genes, the *Wnt* [relating to the *Drosophilia* segmented polarity gene wingless (*Wg*)] and fibroblast growth factor (*Fgf*) family of genes represent major targets of mutagenic effects (36, 39–41). However, the frequency of gene activation responsible for mammary tumor development is dependent on both the strain of the mouse and the virus.

### History of endogenous *Mtv*

Retrospectively, evidence of *Mtv* Sag expression was first observed in experiments demonstrating non-reciprocal lymphocyte activation/proliferation in mixed lymphocyte cultures from MHC-identical strains of mice (42). Before it was known that *Mtv* Sag was the culprit, the antigens responsible for inducing lymphocyte activation/proliferation were termed minor lymphocyte stimulating (*Mls*) antigens. For example, *Mls^a^* expressed by DBA/2 mice results in T cell activation of BALB/c splenocytes when co-cultured. Once monoclonal antibodies to the Vβ regions of the TCR were made available, it became apparent that *Mls^a^* resulted in the activation of BALB/c T cells expressing Vβ6 and that Vβ6^+^ T cells were largely absent in the repertoire of DBA/2 mice. Subsequent genetic studies linked various *Mls* antigens to *Mtv* loci (43–46) and Sag as the element responsible for Vβ-specific T cell interaction (14, 15). Therefore, an important consequence of *Mtv* Sag expression is the ultimate alteration of the peripheral adaptive T cell repertoire mediated by intrathymic deletion of Sag-reactive T cells, the extent and kinetics of which vary depending on the specific Sag (47–49). For example, the Sag associated with *Mtv-7* stimulates and deletes T cells bearing Vβ 6, 7, 8.1, and 9 (50, 51), while that of *Mtv-9* induces complete deletion of TCR Vβ5, 11, and 12 (44, 46, 52). Table 2 provides the chromosomal location and TCR Vβ specificity/deletion for a few of the more commonly encountered endogenous *Mtv* among different mouse strains (53, 54).

**Table 2:** **TCR Vβ specificity and deletion by endogenous *Mtv***.

*Mtv provirus*	Chromosome	TCR Vβ specificity/deletion
*Mtv-1*	7	3
*Mtv-2*	18	14, 15
*Mtv-3*	11	3, 17
*Mtv-6*	16	3, 5, 17
*Mtv-7*	1	6, 7, 8.1, 9
*Mtv-8*	6	11, 12
*Mtv-9*	12	5, 11, 12, 17
*Mtv-11*	14	11, 12, 17
*Mtv-13*	4	3, 17

*This list is not comprehensive of all *Mtv**.

The fact that endogenous retroviruses in general, and *Mtv* in specific remain present in high percentage within the host genome over a long portion of evolution would provide the opportunity for interaction with host genes and subsequent influence of cellular function. Although a large portion of such proviruses no longer encode for functional products, thereby supporting the idea that these endogenous retroviruses are simply genomic “fossils,” there do exist viral elements that have retained activity. The question is therefore what is the evolutionary role or advantage for maintaining these proviruses within the genome? One hypothesis that has been proposed is that *Mtv* are retained by certain mouse strains to serve as an evolutionary means of protection against exogenous milk-borne MMTV infection and MMTV-induced mammary tumors (55). This protection would result from the deletion of *Mtv*-encoded Sag-specific T cells and the subsequent loss of a reactive pool of T cells necessary for infection. In support of this idea, the transgenic expression of the Sag gene of the C3H strain of MMTV has been shown to protect against exogenous MMTV encoding the same Sag specificity (26). However, it is unlikely that this is the only evolutionary role for endogenous *Mtv*, since wild-derived mice lacking *Mtv* are not overwhelmed by mammary carcinomas. Therefore, the question remains, what evolutionary advantage does the maintenance of these endogenous proviral genes impart to the mouse? A number of models have demonstrated that *Mtv* have the capacity to modulate the immune response, thereby providing either a selective disadvantage or advantage in regards to cancer, infection, and immunity. Some of these models require Sag expression and some may require other components of the proviral genome via mechanisms that are not fully understood.

### Influence of endogenous *Mtv* on cancer

Evidence exists that *Mtv* can influence the development of MMTV-induced mammary carcinomas via a mechanism that does not require interaction with CD4^+^ T cells expressing a Sag-reactive TCR Vβ region. A number of mouse strains, including GR, C3H, BR6, and R111 have been selectively inbred for a high incidence of mammary tumors due to their transmission of MMTV to offspring via milk. Two of these strains, GR and C3H, additionally contain endogenous copies of *Mtv* that also generate infectious viral particles. Among weanlings of GR mice, the incidence of pregnancy-independent tumors occurs at a similar frequency whether nursed on GR mothers or foster-nursed on MMTV-free mothers (56). *Mtv-2* was later identified as the dominant gene responsible for tumor development as well as responsible for the expression of MMTV within the milk of GR mice. When C3H mice are freed of their corresponding exogenous C3H-MMTV via foster-nursing, mammary tumors still develop, yet with varying incidence and increased latency. This tumor development was determined to be dependent on the expression of *Mtv-1* (10).

Another tumor model where *Mtv* has been shown to be important is infection with Polyomaviruses (PyVs) – a family of small non-enveloped, double-stranded DNA viruses with potent oncogenic capacity to induce epithelial and mesenchymal cell-derived tumors (57). Susceptibility to PyV-induced tumors has been shown among certain strains of H-2^k^ expressing mice (58, 59). A previously identified PyV susceptibility gene (*Pyv^S^*) (60) was later identified to encode the Sag from *Mtv-7* among H-2^k^ mice (C3H/BiDa mice) resulting in the intrathymic deletion of TCR Vβ6 expressing T cell populations (59). Among other mechanisms (61), preferential usage of H-2^k^-restricted CD8^+^ T cells expressing TCR Vβ6 that were specific for an immunodominant peptide derived from the viral middle T protein were demonstrated to provide the necessary antitumor immunity against PyV (62). Susceptibility to PyV is transmitted as a dominant trait due to the requirement of only a single copy of the superantigen being necessary for the deletion of T cells expressing specific Vβ TCRs. H-2^k^-identical C57BR/cdJ (BR mice) retain this cytotoxic CD8^+^ TCR Vβ6 population due to the lack of *Mtv-7* and are therefore highly resistant to tumor development.

*Mtv-7* has also been shown to influence the immune response among H-2^d^-expressing mice in a murine model of aggressive lymphoma. Although DBA/2 and B10.D2 strains of mice are syngeneic at the MHC and therefore immunologically compatible, they demonstrate varied immune response and outcome upon challenge with the highly malignant DBA/2-derived ESb cell line (63). B10.D2 mice are able to prevent metastasis of such tumors by generating and sustaining a sufficient humoral and cellular immune response. This response is evident by the increased sensitivity of B10.D2 following irradiation or depletion of CD4^+^ and CD8^+^ T cells (64). In contrast, naïve DBA/2 mice are highly susceptible to ESb-induced lymphoma (65). The generation of recombinant inbred (RI) mouse strains from the cross of B10.D2 and DBA/2 facilitated the identification of potential genes segregating with ESb-related tumor susceptibility and resistance (63). Genotyping for *Mtv* among several of the tumor-resistant RI strains revealed similarity to the parental DBA/2 strain except for the loss of a particular LTR that corresponded to the *Mtv-7* provirus. It was further demonstrated that ESb tumor cells themselves express proviral *Mtv-7* at both the mRNA and protein level (66). Instead of inducing anergy among the Sag-reactive cells, the ESb tumor-associated *Mtv-7*-encoded Sag was demonstrated to induce activation of Sag-specific cytotoxic T lymphocytes. TCR Vβ6 cells were demonstrated to facilitate specific killing of tumor cells expressing the endogenous *Mtv-7 in vitro*. Furthermore, treatment of tumor-bearing DBA/2 mice with TCR Vβ6 T cells from naïve B10.D2 mice led to a significant increase in survival and concurrent reduction in tumor growth (66). It is important to note that the reduction in tumor growth and delay in death lasted only 10 days, suggesting the potential and eventual loss of the TCR Vβ6 T cell population. Therefore, in models of both PyV and ESb-induced lymphoma, the presence of *Mtv-7* affords a selective disadvantage in terms of deleting a protective population of lymphocytes that are necessary for tumor immunity.

In contrast to the deletion of Sag-reactive T cells, *Mtv*-encoded Sag may further modulate tumor susceptibility via activation of Sag-reactive T cells. Spontaneous reticulum cell sarcoma (RCS), a form of B cell lymphoma, has been observed to arise in >90% of SJL mice older than 12 months of age. The development of such RCS requires the presence of host T cells and specifically that of T cell-derived cytokines, such as IL-5 and IFN-γ (67, 68). The strong proliferative response of previously unsensitized CD4^+^ T cells to RCS cells (69) and the biased usage of such responding cells expressing TCR Vβ16 imply a role for superantigen in this model (70). CD4^+^ TCR Vβ16 cells have been shown to be capable of supporting RCS growth via the production of IL-2, IL-4, and IL-5. Additional analysis revealed the high expression of a novel MMTV-LTR among mRNA from RCS cells (70). It was subsequently determined that the B cell lymphomas of SJL mice contained *Mtv-29*, which via the *Mtv-29-*encoded Sag were capable of stimulating TCR Vβ16 cells that are required to support the development of spontaneous B cell lymphomagenesis (71).

Although a majority of the research examining the role of *Mtv* in modulation of the immune response has mainly focused on Sag-dependent processes, evidence does exist to support *Mtv-*associated, Sag-independent mechanisms. BALB/c mice harbor three distinct *Mtv* (*Mtv-6, -8*, and *-9*) all on separate chromosomes, which in the context of MHC class II I-E induce intrathymic deletion of reactive T cells expressing TCR Vβ3, 5, 11, and 12. The generation of BALB/c congenic mice lacking all endogenous *Mtv* (BALB/*Mtv*-null) enabled the unique opportunity to examine the influence of *Mtv* on host response to a variety of models (72). In the absence of endogenous *Mtv*, the incidence of mammary tumors in response to exogenous MMTV infection was reduced from 100% (in BALB/c) to 10% (in BALB/*Mtv*-null). This drastic reduction in tumor incidence was independent of the infection route, whether milk-borne infection or following intraperitoneal injection of a stable cell line expressing the infectious cloned MMTV provirus. Unlike some mouse strains that demonstrate resistance to exogenous MMTV infection via the production of neutralizing antibodies (73), such was not the case with the BALB/*Mtv*-null mice. BALB/*Mtv*-null mice also demonstrated resistance to infection with the MMTV variant type B leukemogenic virus (TBLV) (74), which due to a truncated Sag protein lacks the ability to induced Sag-mediated T cell deletion and thereby induces T cell lymphoma rather than MMTV-induced mammary cancers.

The C3H strain of MMTV that was shown to have reduced capacity to induce tumorigenesis in BALB/*Mtv*-null mice (72) encodes a weak Sag resulting in a slow and marginal deletion of CD4^+^ T cells expressing the C3H Sag-reactive TCR Vβ14 (48). This would suggest that endogenous *Mtv*-encoded Sag are necessary during the early stages of infection for exogenous Sag presentation and cognate T cell deletion. In contrast to C3H-MMTV, infection with FM-MMTV, which encodes a stronger Sag (75, 76) and therefore results in a much larger response by the cognate TCR Vβ8.2^+^ population, resulted in a similar magnitude and kinetics of Sag response between BALB/c and BALB/*Mtv*-null mice. These findings imply that the extent of deletion associated with the exogenous MMTV-encoded Sag dictates the impact of endogenous Sag during MMTV infection. While endogenous Sag appear essential during the early stages of infection with C3H-MMTV, a stronger Sag such as the FM-MMTV strain can overcome this requirement in terms of establishing the initial infection. Regardless of this early and initial infectivity, the absence of *Mtv* in BALB/*Mtv*-null mice affords them high resistance to FM-MMTV-induced tumorigenesis and such mice are further incapable of transmitting the virus via their milk (76).

### Influence of endogenous *Mtv* on infection

In the experimental murine model of *Leishmania major* infection, differences in susceptibility have been attributed to the host’s ability to generate a sufficient Th1-biased, IFN-γ-dominated, CD4^+^ T cell response (77). This is most evident upon analysis of the highly susceptible and Th2/IL-4-prone BALB/c strain compared to the highly resistant C57BL/6 strain that is prototypical of a Th1/IFN-γ response. Although certainly much more resistant to disease compared to the BALB/c strain, the CBA/CaJ and CBA/J strains of mice differ in their responses to *L. major* infection. In contrast to CBA/CaJ mice that develop only transient lesions upon infection, persistent inflammatory lesions and 10-fold higher parasite density are observed in CBA/J mice (78). A quantitative reduction in the production of IFN-γ in the CBA/J was determined to account for the persistent lesions upon infection. This reduction in cytokine production further correlated with the presence of *Mtv-7* in the CBA/J strain that is absent in the CBA/CaJ mice. However, unlike the above models of PyV and ESb-induced lymphoma, *Mtv-7*-specific TCR Vβ6 T cells do not constitute the predominant responding population during *L. major* infection. Furthermore, the overall magnitude and heterogeneity of the CD4^+^ T cell response was similar between the two strains (78). Such findings would suggest that T cells in general, regardless of their TCR Vβ repertoire commit somewhat poorly to a Th1-biased, IFN-γ-producing subset in the presence of *Mtv-7* during leishmaniasis. This *L. major*-specific effect in the presence of *Mtv-7* was determined to be mediated via alteration of the T cell priming ability of antigen presenting cells. Although no difference was observed in *L. major*-infected, bone marrow-derived dendritic cells (BMDCs) between CBA/CaJ and CBA/J in terms of their expression of various cell surface and co-stimulatory markers, CD4^+^ T cells from CBA/J mice did produce increased IFN-γ if primed in the presence of *L. major*-infected BMDCs from CBA/CaJ mice compared to their own BMDCs (78). Since impairment of the CBA/J immune response is not due to a specific TCR Vβ subset, these findings would suggest a *Mtv-7*-dependent, Sag-independent mechanism responsible for modulating T cell priming during the pathogenesis of leishmaniasis.

The murine model of *Plasmodium berghei* ANKA infection constitutes a widely used model that reflects some experimental similarities to that of human cerebral malaria (CM). The neurological symptoms of genetically susceptible mice infected with *P. berghei* ANKA have been shown to be immune mediated, mainly dependent on CD4^+^ and CD8^+^ T cells (79, 80). In this model, the high production of Th1-derived cytokines, including IFN-γ and TNF-α, via interferon regulatory factor-1 (IRF-1) has been implicated in malarial neuropathogenesis (81, 82). The peripheral expansion of a restricted T cell population expressing TCR Vβ8.1 and Vβ8.2 was observed in the highly susceptible B10.D2 (H-2^d^) strain that manifest severe neurological symptoms but not in infected mice that failed to develop CM. The occurrence of CM was significantly reduced following treatment with a monoclonal antibody against TCR Vβ8.1 specific T cells. Furthermore, the endogenous presence of *Mtv*-*7* in congenic BALB.D2 mice, or the exogenous counterpart MMTV-SW that encodes the same Sag as *Mtv-7* and is present in BALB.SW mice, results in Sag-mediated deletion or anergy of a variety of Vβ-specific populations including Vβ8.1 that has been demonstrated to provide protection against CM (83). To investigate whether the presence of *Mtv-7* was sufficient to induce resistance to CM, RI strains derived from a cross between the susceptible BALB/c (negative for *Mtv-7*) and resistant DBA/2 (positive for *Mtv*-*7)* were examined for their response during *P. berghei* ANKA infection. A strong correlation was identified between the presence of *Mtv*-*7* and protection against neuropathogenesis of CM (84). The similar levels of parasitemia observed between *Mtv*-*7*^+^ and *Mtv*-*7^−^* mice would suggest that integration of the viral genome did not have any effect on at least the initial stages of parasite growth and development (84). Overall, it was concluded that deletion of T cells expressing TCR Vβ8.1 among H-2^d^ expressing strains of mice, whether via the exogenous or endogenous presence of *Mtv-7*-encoded Sag was sufficient to confer resistance to CM. Unlike the models of PyV, ESb-induced lymphoma, and *L. major* infection, in which the presence of *Mtv* confers a selective disadvantage to the host and increases susceptibility, *Mtv* appears to provide protection during the neuropathogenesis of *P. berghei* ANKA infection, by an as of yet unidentified mechanism.

In addition to modulation of the conventional population of CD8^+^ and CD4^+^ T cells, *Mtv* have been demonstrated to influence the immune system during viral infection via expansion of a particular subset of CD4^+^ T cells expressing the transcription factor Foxp3. Murine infection with the clone 13 isolate of lymphocytic choriomeningitis virus (LCMV) represents a model of chronic viral infection, in which T cell exhaustion results in infection virtually for life (85, 86). Infection of mice with LCMV clone 13 resulted in the selective expansion of a TCR Vβ specific population of Foxp3^+^ regulatory T cells (Treg) (87). The expansion of TCR Vβ5^+^ Treg was determined to be MHC class II-dependent, CD4-independent, and secondary to stimulation by the *Mtv-9*-encoded Sag. Treg play a key role in immune suppression and control of organ-specific autoimmune diseases via a variety of mechanisms. Whether this specific *Mtv*-encoded Sag-mediated expansion of Treg influences the pathogenesis of viral or other infectious agents remains to be determined. Nevertheless, it does demonstrate the wide range of influence *Mtv* has on the immune system, influencing both effector and suppressor lymphocyte populations.

In addition to the reduction in tumorigenicity via both a Sag-dependent and Sag-independent manner, the absence of *Mtv* provided further resistance to inoculation with the gram-negative bacterium *Vibrio cholerae*, as demonstrated by decreased bacterial replication and mortality in BALB/*Mtv*-null mice. However, the absence of *Mtv* did not provide absolute resistance to bacterial challenge, as infection with *Salmonella typhimurium* resulted in similar mortality between BALB/c and BALB/*Mtv*-null mice. The reintroduction of any single *Mtv* provirus into the BALB/*Mtv*-null mice (generating congenic mice positive for either *Mtv-6, -8*, or *-9*) was sufficient to restore susceptibility to both select viral (MMTV-induced mammary tumors) as well as bacterial (*V. cholera*) challenge. Interestingly, the difference in susceptibility to *V. cholera* was observed as early as 48 h following infection, suggesting *Mtv* may function to regulate processes of innate immunity. However, the exact mechanism for this increased susceptibility to disparate pathogens among mice expressing at least one or more *Mtv* remains to be elucidated. One clue may resolve around the unique fact that unlike *Mtv-8* and *-9, Mtv-6* lacks a >6 kb portion of sequence encoding most of the viral Gag, Env, and Pol proteins. *Mtv-6* does encode functional Sag, which would suggest that a Sag-mediated or as of yet unidentified *Mtv*-encoded product may modulate an immune response that is uniquely shared between MMTV and *V. cholera*.

### Influence of endogenous *Mtv* on immunity

In addition to the above models, *Mtv-7* was implicated to modulate the immune response during the progression of graft-versus-host disease (GVHD). In this model, the transfer of either parental C57BL/6 or DBA/2 lymphoid cells into F1 offspring (B6D2F1) yields opposing disease outcome. Donor lymphoid cells from C57BL/6, which are associated with a Th1-biased, IFN-γ-mediated cytokine response, induce an acute form of GVHD. On the other hand, the Th2-prone, IL-4-mediated response characteristic of DBA/2 yields a more chronic disease. Although C57BL/6 and DBA/2 differ in MHC haplotype, such a difference between the induction of an acute and chronic response was attributed to a non-MHC-related mechanism (88). *Mtv-7*, or that of a closely linked locus to *Mtv-7* was implicated in the relative development of acute and chronic GVDH based on data from congenic C57BL/6 mice containing DBA/2-derived alleles at the region surrounding *Mtv-7* on chromosome 1 (89). When lymphoid cells from the congenic C57BL/6 (H-2^d^) were transferred to recipient B6D2F1 mice, the mice developed signs more consistent with that of chronic GVDH than that of the acute disease normally associated after transfer of C57BL/6 cells (89). Furthermore, transfer of the congenic C57BL/6 lymphoid cells containing the DBA/2-derived alleles around *Mtv-7* resulted in decreased expression levels of IFN-γ and increased levels of IL-4 compared to the control C57BL/6 transfer. Although these data would suggest a *Mtv-7*-mediated influence on disease outcome, depletion of *Mtv-7*-encoded Sag-reactive cells from the donor population did not impact the development of acute GVHD. As such, the possibility remains that a locus near that of *Mtv-7* on chromosome 1 can influence host immune response and the eventual outcome of GVHD.

**Figure 1:**
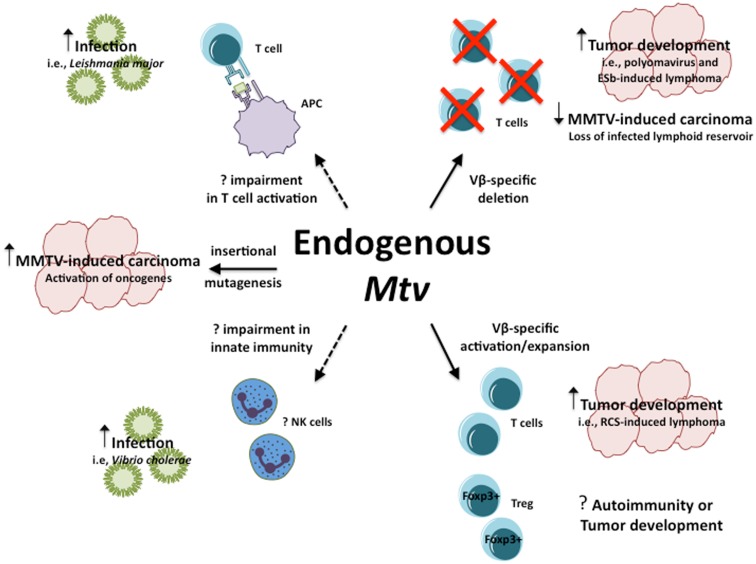
**Influence of endogenous *Mtv* on host response to infection and disease**. Potential mechanisms by which endogenous *Mtv* influence host response during infection and disease. Solid lines represent known mechanisms, dashed lines represent potential mechanisms. Question marks represent outcomes that remain to be determined.

Collectively, the above models demonstrate the range of influence endogenous *Mtv* have in their ability to modulate host susceptibility to a variety of infections and diseases in seemingly opposite manners (Figure 1). In the case of PyV infection and ESb-induced lymphoma, *Mtv*-encoded Sag induce deletion of tumor-reactive T cells thereby facilitating tumor development and progression. At the same time, *Mtv*-mediated activation/stimulation of Sag-reactive T cells appears to promote neuropathogenesis during *P. berghei* ANKA infection as well as induce B cell lymphoma. In addition to conventional T cells, *Mtv* has been shown to modulate the population of Foxp3^+^ regulatory T cells via a Sag-dependent manner during the course of chronic viral infection. The activation of Treg can combat and limit the extent of autoimmune disease while at the same time facilitate tumor progression by suppressing tumor immunity. The potential ability to modulate such a dynamic population of T cells can therefore have important implications on host outcome. In addition to Sag-dependent mechanisms, other as of yet unidentified genes from the *Mtv* provirus may be capable of influencing Sag-independent immune responses, such as during *L. major* infection. Furthermore, *Mtv*-mediated influence may not be restricted to the adaptive immune system, as evidence using BALB/*Mtv*-null mice may suggest modulation of potential innate immune responses.

## Conclusion

The co-evolution of *Mtv* and its murine host has allowed ample time for the virus to develop various mechanisms to modulate immune regulatory mechanisms. While many endogenous proviral gene sequences have acquired mutations that have led them to be non-functional, Sag expression has remained intact suggesting some benefit for the host. Recent advances suggest that these advantages go beyond the initial idea that *Mtv* exist solely as a way to prevent exogenous MMTV-induced mammary carcinoma. While the exact mechanisms by which *Mtv* function in all of these various models are not fully known, further insight into how endogenous *Mtv*, and in particular *Mtv* Sag, influence the immune system can provide potential benefit. Such knowledge may prove useful for therapeutic modifications of lymphocytes using retroviral-based vectors for gene therapy (90). In addition, although there are differences in terms of the activity of viral and bacterial superantigens, the knowledge gained from endogenous retroviruses may prove potentially useful for the targeting of Sag-reactive lymphocytes to tumor cells (91). Ultimately, understanding the mechanisms underlying retroviral integration and interaction with the immune system could provide benefits to a multitude of disease processes, including infectious diseases and cancers.

## Conflict of Interest Statement

The authors declare that the research was conducted in the absence of any commercial or financial relationships that could be construed as a potential conflict of interest.
